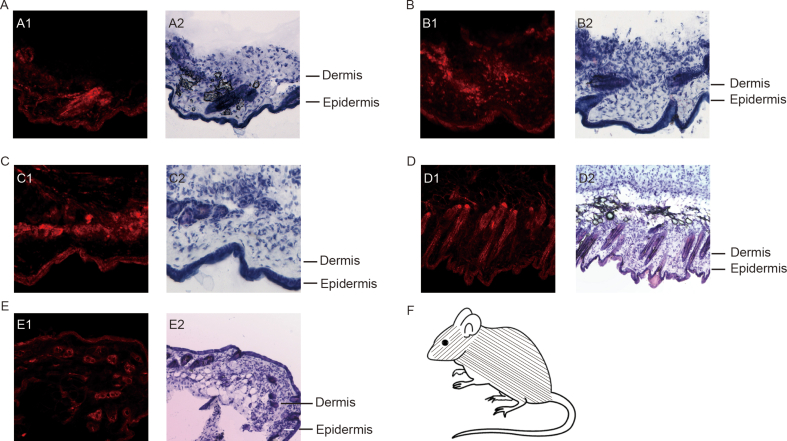# MAFA-expressing panniculus carnosus regulates skin twitching in mice

**DOI:** 10.1016/j.gendis.2025.101975

**Published:** 2025-12-11

**Authors:** Junqi Jia, Xinyue Mei, Yixiu Liu, Yang Yan, Guang Yu, Lan Zhou, Changming Wang

**Affiliations:** School of Medicine, Nanjing University of Chinese Medicine, Nanjing, Jiangsu 210023, China

MAFA is a transcription factor expressed in the pancreas and dorsal root ganglion neurons. The panniculus carnosus, also referred to as the cutaneous trunk muscle, cutaneous trunci, or cutaneous maximus, is a cutaneous muscle situated in the deep layer of human skin. We developed a MAFA-cre mouse and employed morphological and optogenetic techniques to investigate MAFA expression in the skin and its associated functions. Our novel findings indicate that MAFA is expressed in the panniculus carnosus muscles, and activation of these MAFA-expressing muscles induces twitching in the back.

To further explore the function of MAFA, we generated MAFA-cre mice as we previously described.^1^ To elucidate the expression pattern of MAFA in the skin, we crossed MAFA-cre mice with ROSA26 mice to produce MAFA-tdTomato mice (Fig. 1A). Our previous research has demonstrated that fibers of MAFA-positive dorsal root ganglion neurons are distributed in both hairy and glabrous skin.^1^ In this study, we observed extensive MAFA expression in the deep layer of skin, including that of the back, cheek, and head (Fig. 1B1–D1, B2–D2). To validate the structural integrity of these skin samples, we performed hematoxylin-eosin staining (Fig. 1B3–D3, B4–D4). Notably, we discovered that MAFA expression is strongly localized within the skin's muscle layer. The muscle within the skin is commonly referred to as the panniculus carnosus. Consequently, we determine that MAFA is expressed in the panniculus carnosus within the skin.

**Figure 1:**
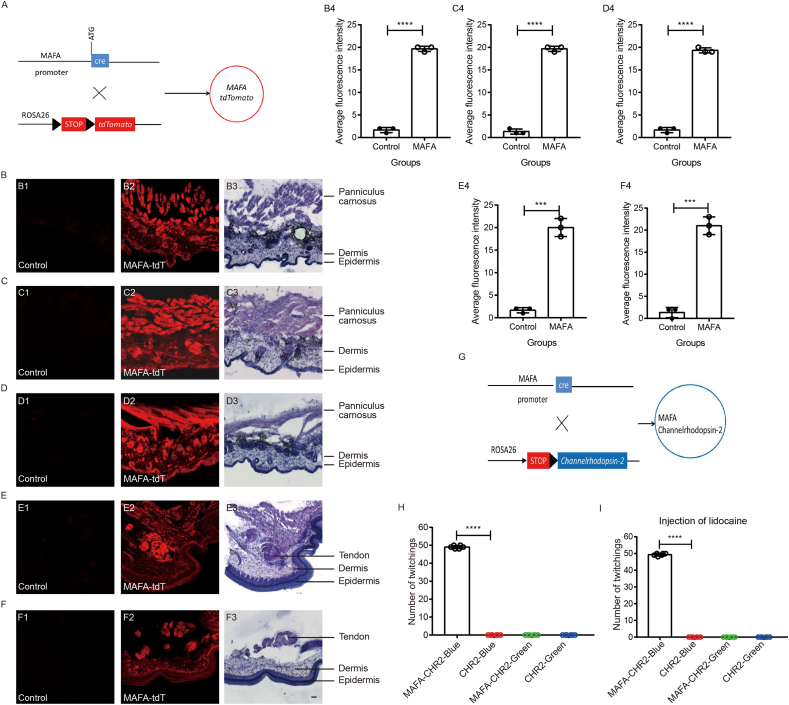
MAFA-positive panniculus carnosus activation induced skin twitching in mice. **(A)** Mating strategy of the MAFA-tdTomato mouse. **(B)** MAFA expression in back skin (B1: MAFA-CRE mice without tdT as negative control; B2: MAFA-tdT mice) and hematoxylin-eosin staining of the back skin (B3). (B4) Average fluorescence intensity of MAFA-tdT in the mouse skin. Images in both groups were captured with the same settings (gain and laser power). *n* = 3 mice/group. **(C)** MAFA expression in check skin (C1: MAFA-CRE mice without tdT as negative control; C2: MAFA-tdT mice) and hematoxylin-eosin staining of the check skin (C3). (C4) Average fluorescence intensity of MAFA-tdT in the mouse skin. Images in both groups were captured with the same settings (gain and laser power). *n* = 3 mice/group. **(D)** MAFA expression in head skin (D1: MAFA-CRE mice without tdT as negative control; D2: MAFA-tdT mice) and hematoxylin-eosin staining of the head skin (D3). (D4) Average fluorescence intensity of MAFA-tdT in the mouse skin. Images in both groups were captured with the same settings (gain and laser power). *n* = 3 mice/group. **(E)** MAFA expression in palm skin (E1: MAFA-CRE mice without tdT as negative control; E2: MAFA-tdT mice) and hematoxylin-eosin staining of the palm skin (E3). (E4) Average fluorescence intensity of MAFA-tdT in the mouse skin. Images in both groups were captured with the same settings (gain and laser power). *n* = 3 mice/group. **(F)** MAFA expression in plantar skin (F1: MAFA-CRE mice without tdT as negative control; F2: MAFA-tdT mice) and hematoxylin-eosin staining of the plantar skin (F3). (F4) Average fluorescence intensity of MAFA-tdT in the mouse skin. Images in both groups were captured with the same settings (gain and laser power). *n* = 3 mice/group. Scale bar: 50 μm. **(G)** Mating strategy of MAFA-Channelrhodopsin-2 mouse. **(H)** The number of twitchings in 10 s in MAFA-cre-CHR2 mice and CHR2 mice after optogenetic stimulation with a blue laser or green laser (negative control). *n* = 6. **(I)** The number of twitchings in 10 s in MAFA-cre-CHR2 mice and CHR2 mice by optogenetic stimulation with a blue laser or green laser (negative control) after intracutaneous injection of lidocaine. *n* = 6. ∗∗∗*P* < 0.001 and ∗∗∗∗*P* < 0.0001. Data were analyzed using two-tailed Student's *t*-test (B4, C4, D4, and F4) and two-way ANOVA with Bonferroni's post hoc test (H, I).

Although the panniculus carnosus has largely degenerated in humans, remnants of it persist. In this study, we elucidated the distribution of the panniculus carnosus by examining MAFA expression in mice. Various regions of the mouse body exhibited MAFA-positive panniculus carnosus. However, MAFA-positive panniculus carnosus was not observed in the palmar and plantar regions of the skin, although some MAFA-positive tendons were detected in deeper tissues (Fig. 1E and F). Our findings indicated an absence of MAFA-positive panniculus carnosus in the chest (Fig. S1A), dorsal part of hind paw (Fig. S1B), dorsal part of front paw (Fig. S1C), arm (Fig. S1D), and leg (Fig. S1E). In summary, MAFA-positive panniculus carnosus is distributed in the skin of the back, cheek, and head (Fig. S1F).

Currently, no abnormal physiological functions have been identified in MAFA-knockout (MAFA-KO) mice. To investigate the function of MAFA-positive panniculus carnosus in mice, we employed an optogenetic stimulation approach to activate MAFA. We generated MAFA-Channelrhodopsin-2 mice (Fig. 1G) and subjected them to laser stimulation. Unexpectedly, we observed a twitching behavior upon stimulating the shaved skin on the back (Fig. 1H; Video 1–4). Specifically, light stimulation rapidly induced this twitching behavior. To ascertain whether the panniculus carnosus was responsible for the twitching, we administered a local injection of lidocaine into the skin muscle. Notably, the twitching induced by blue laser stimulation in MAFA-cre-CHR2 mice remained unaffected by lidocaine (Fig. 1I). These findings suggest that the activation of the panniculus carnosus expressing MAFA induces twitching in mice.

Supplementary video related to this article can be found at https://doi.org/10.1016/j.gendis.2025.101975

The following is/are the supplementary data related to this article.

Video 1 **Videos of skin twitching by activation of the MAFA-positive panniculus carnosus muscle in the back.**
MAFA-cre-CHR2 mice were stimulated by the optogenetic stimulation (blue laser).2

Video 2 CHR2 mice were stimulated by the optogenetic stimulation (blue laser).3 Video 2

Video 3 MAFA-cre-CHR2 mice were stimulated by the optogenetic stimulation (green laser, negative control).4 Video 3

Video 4 CHR2 mice were stimulated by the optogenetic stimulation (green laser, negative control).5 Video 4

MAFA is a transcription factor known to bind to the insulin enhancer and activate insulin gene expression.^2^^,^^3^ Previous research has identified MAFA expression in the dorsal root ganglion of chicks and in low-threshold mechanoreceptors.^4^^,^^5^ Recently, we developed MAFA-cre mice to investigate its function further.^1^ To our surprise, we discovered strong MAFA expression in the skin. To confirm this expression, we employed hematoxylin-eosin staining methods on the skin. We identified a thin layer of muscle, typically referred to as the panniculus carnosus, exhibiting strong MAFA expression.

Using an optogenetic stimulation method, we observed twitching behavior in the back of the mouse. The onset of skin twitching was observed immediately following stimulation with a blue laser. Based on our previous study, it is known that the fibers of MAFA-positive neurons innervate hairy skin.^1^ We hypothesized that the twitching was induced by the stimulation of MAFA-positive nerve endings. Additionally, it was considered that the activation of MAFA-positive panniculus carnosus, stimulated by the laser, could also be responsible for the twitching behavior. To test this hypothesis, lidocaine was locally injected into the panniculus carnosus. Our findings indicated that the twitching behavior remained unaffected by lidocaine, suggesting that the activation of MAFA-expressing panniculus carnosus directly induces the twitching.

Historically, Turner (1870) described this cutaneous muscle as a vestigial form of the panniculus carnosus. Currently, several functions are attributed to the panniculus carnosus, which may imply the role of the MAFA-positive panniculus carnosus. Primarily, the panniculus carnosus may facilitate the movement of the skin over deeper musculature or enable skin “twitching” to dislodge insects. Our research shows that the activation of MAFA-expressing panniculus carnosus indeed induces twitching behavior. We hypothesize that this phenomenon may be related to the repulsion of insects from the mice, although our mice were bred under specific pathogen-free conditions. The MAFA-positive panniculus carnosus in the head is also important for the rodent whisker movement. Secondly, the panniculus carnosus, located near the wound edge, may expedite healing. The activation of MAFA, which induces twitching behavior, might facilitate wound healing. Thirdly, it is proposed that the MAFA-expressing panniculus carnosus generates heat through contraction, akin to shivering in humans when cold. Although we did not observe a temperature increase when stimulating the mice using an optogenetic method, the twitching behavior may serve to preserve body heat. Finally, we speculate that the twitching behavior induced by MAFA activation may help alleviate pain or itch. These speculations suggest a protective function of the MAFA-positive panniculus carnosus. However, the specific functions of MAFA in the panniculus carnosus remain unclear, necessitating further research to elucidate its role.

There are mainly two reasons for the absence of panniculus carnosus in the chest, dorsal part of hind paw, dorsal part of front paw, arm, or leg in mice. Firstly, it may be due to the evolutionary adaptation. In the process of evolution, with the emergence of upright walking and the liberation of upper limbs, the movement mode and functional requirements of the body have changed. The movement of the chest and limbs is more complete by bones, joints, and deep muscles, and the role of skin muscles is relatively weakened, so it gradually degenerates and its distribution decreases. Secondly, the skin of the chest and limbs is mainly realized by other structures and muscles. The deep muscles have gradually assumed more motor functions in evolution, which has reduced the importance of skin muscles.

Furthermore, we also showed MAFA to have strong expression in the tendon, which hints at its important function in motor regulation. The role of MAFA in movement remains largely unexplored.

In this study, we have identified a novel expression of MAFA in the panniculus carnosus and observed the phenomenon of twitching induced by the activation of MAFA-positive panniculus carnosus. The finding has broad implications for neuromuscular physiology, wound healing, thermoregulation, and sensory disorders, positioning MAFA as a potential therapeutic target in cutaneous and motor pathologies.

## CRediT authorship contribution statement

**Junqi Jia:** Methodology, Data curation. **Xinyue Mei:** Methodology, Data curation. **Yixiu Liu:** Methodology, Data curation. **Yang Yan:** Project administration. **Guang Yu:** Project administration. **Lan Zhou:** Project administration. **Changming Wang:** Conceptualization, Writing – original draft, Funding acquisition, Validation, Writing – review & editing.

## Data availability

The raw data supporting the conclusions of this article will be made available by the authors, without undue reservation.

## Ethics statement

This study was approved by the Animal Care and Use Committee of Nanjing University of Chinese Medicine (Nanjing, China). Experiments were conducted according to the animal research ethical guidelines of the International Association for the Study of Pain.

## Funding

This work was supported by the 10.13039/501100001809National Natural Science Foundation of China (No. 82474211 to C.M.W.), the Development Plan of Traditional Chinese Medicine Science and Technology in Jiangsu Province, China (MS2024002, C.M.W.), and a Project Funded by the Priority Academic Program Development of Jiangsu Higher Education Institutions (Integrated Traditional Chinese and Western) (China).

## Conflict of interests

The authors have no conflict of interests to disclose.

## Figures and Tables

**Supplementary figure S1:**