# Supplementary material for: MAFA-expressing panniculus carnosus regulates skin twitching in mice
Source: Genes Dis. 2025 Dec 11;13(4):101975. doi: 10.1016/j.gendis.2025.101975 (PMC12989827; doi:10.1016/j.gendis.2025.101975)
Supplement: Multimedia component 1 [file mmc1.docx]

**Material and methods**

**Animals**

C57Bl/6J and *MAFA-Cre* mice were bred under specific pathogen-free conditions in the Johns Hopkins University School of Medicine Animal Facility and weaned at 3-3.5 weeks of age. Mice were kept in community cages (4-5 mice per cage) at light periods of 12 h and fed water and mouse chow ad libitum. Male mice were used for experiments at 8-10 weeks of age. All animal procedures were conducted in accordance with institutional guidelines and with approval from the Animal Care and Use Committee at the Johns Hopkins University School of Medicine.

**Generation of MAFA^cre+/-^ mice**

We inserted the Cre just upstream of the MAFA ATG, but replicate the few nucleotides before the ATG so that the context of the ATG resembles that of the native MAFA locus. This will result in an unmodified upstream sequence and unmodified Cre coding sequence.

**Immunofluorescence of skin**

MAFA-tdTomato mice were anesthetized with chloral hydrate and transcardially perfused with PBS and ice-cold 4% PFA. The skin of the back, cheek, trunk, palm, plantar, et, al were dissected. After being washed in PBS, tissues were equilibrated sequentially in 15% and 30% sucrose, embedded in optimum cutting temperature compound, and sectioned with a cryostat. The sections on slides were dried at 37℃ for 1 h, and fixed with 4% PFA at RT for 10 min. The slides were pre-incubated in blocking buffer (10% normal goat serum, 0.2% Triton-X-100 in PBS, pH 7.4) for 1 h at RT. Sections were washed three times with PBS and fluoromount (Southern Biotech) was applied before coverslips were placed over section.

**HE staining**

The adjacent slides of immunofluorescence experiments was used for HE staining. After being washed with PBS, tissues was equilibrated sequentially in 15% and 30% sucrose, embedded in optimum cutting temperature compound, and sectioned with a cryostat. A HE staining kit (H3502, Vector Lab, USA) was used. The protocols were according to the manufacturer's instructions. Absolute ethanol for 5 min, 95% ethanol for 2 min, 80% ethanol for 2 min, 70% ethanol for 2 min, distilled water 2 min. Dyeing with hematoxylin solution for 5-20 min, differentiation fluid 30 s, and immersed with tap water for 15 min. Eosin staining for 30s-2min, immersed with tap water for 5min. They were sealed with neutral gum and observed under microscopy (Axio Zoom.V16, Zeiss, Germany).

**Optogenetic stimulation**

Mice were shaved on the back one day before the optogenetic stimulation. MAFA-Channelrhodopsin-2 mice were used for optogenetic stimulation, a 50-Hz blue laser or green laser (Negative control) was applied to the back through the transparent glass for 1 min after the mice were anesthetized by isoflurane.

**Local injection of lidocaine**

4 sites for injection of lidocaine hydrochloride (2%, Merck, USA) in the shaved back of mice were selected. 20 μl of lidocaine in each site was intradermal injected. The optogenetic stimulation experiments were started immediately, 30 minutes and 60 minutes after lidocaine injection.

**Statistical analysis**

All statistical data are reported as the standard error of the mean (SEM), along with the number of samples analyzed (n). Statistical analyses were conducted using Student’s t-tests and/or analysis of variance (ANOVA), followed by appropriate post hoc tests. A p-value of less than 0.05 was considered statistically significant.
